# Joint modelling of time-to-clinical malaria and parasite count in a cohort in an endemic area

**DOI:** 10.7243/2053-7662-7-1

**Published:** 2019

**Authors:** Christopher C. Stanley, Lawrence N. Kazembe, Andrea G. Buchwald, Mavuto Mukaka, Don P. Mathanga, Michael G. Hudgens, Miriam K. Laufer, Tobias F. Chirwa

**Affiliations:** 1 School of Public Health, Faculty of Health Sciences, University of the Witwatersrand, Johannesburg, South Africa.; 2 Malaria Alert Center, University of Malawi College of Medicine, Blantyre, Malawi.; 3 Department of Statistics, University of Namibia, Windhoek, Namibia.; 4 Center for Vaccine Development and Global Health, University of Maryland School of Medicine, Baltimore, USA.; 5 Oxford Centre for Tropical Medicine and Global Health, Oxford, United Kingdom.; 6 Mahidol-Oxford Tropical Medicine Research Unit, Bangkok, Thailand.; 7 Department of Biostatistics, Center for AIDS Research (CFAR), University of North Carolina Chapel Hill, North Carolina, USA.

**Keywords:** Prospective studies, longitudinal data, malaria parasite, time-to-event, clinical malaria, Cox proportional hazards model, joint modelling

## Abstract

**Background:**

In malaria endemic areas such as sub-Saharan Africa, repeated exposure to malaria results in acquired immunity to clinical disease but not infection. In prospective studies, time-to-clinical malaria and longitudinal parasite count trajectory are often analysed separately which may result in inefficient estimates since these two processes can be associated. Including parasite count as a time-dependent covariate in a model of time-to-clinical malaria episode may also be inaccurate because while clinical malaria disease frequently leads to treatment which may instantly affect the level of parasite count, standard time-to-event models require that time-dependent covariates be external to the event process. We investigated whether jointly modelling time-to-clinical malaria disease and longitudinal parasite count improves precision in risk factor estimates and assessed the strength of association between the hazard of clinical malaria and parasite count.

**Methods:**

Using a cohort data of participants enrolled with uncomplicated malaria in Malawi, a conventional Cox Proportional Hazards (PH) model of time-to-first clinical malaria episode with time-dependent parasite count was compared with three competing joint models. The joint models had different association structures linking a quasi-Poisson mixed-effects of parasite count and event-time Cox PH sub-models.

**Results:**

There were 120 participants of whom 115 (95.8%) had >1 follow-up visit and 100 (87.5%) experienced the episode. Adults >15 years being reference, log hazard ratio for children <5 years was 0.74 (95% CI: 0.17, 1.26) in the joint model with best fit vs. 0.62 (95% CI: 0.04, 1.18) from the conventional Cox PH model. The log hazard ratio for the 5–15 years was 0.72 (95% CI: 0.22, 1.22) in the joint model vs.0.63 (95% CI: 0.11, 1.17) in the Cox PH model. The area under parasite count trajectory was strongly associated with the risk of clinical malaria, with a unit increase corresponding to-0.0012 (95% CI: −0.0021, −0.0004) decrease in log hazard ratio.

**Conclusion:**

Jointly modelling longitudinal parasite count and time-to-clinical malaria disease improves precision in log hazard ratio estimates compared to conventional time-dependent Cox PH model. The improved precision of joint modelling may improve study efficiency and allow for design of clinical trials with relatively lower sample sizes with increased power.

## Introduction

Malaria remains one of the most common parasitic infections globally with a disproportionately high burden in sub-Saharan Africa [1]. In malaria-endemic areas, repeated exposure results in acquired immunity to clinical malaria disease but not infection. Of interest in many malaria studies is to estimate time-to-clinical malaria but repeated exposure may give rise to a relationship between the disease and time-dependent covariates such as parasite count. However, time-to-clinical malaria and parasite count data are often analysed separately, mostly using Cox proportional hazards (PH) models and mixed-effects models or generalised estimating equations (GEE) respectively [2–4]. Separate analysis of time-to-clinical malaria disease and parasite count data may result in inefficient estimates when these two processes are strongly associated [5].

For accurate estimation of the risk of clinical malaria, analytical methods are required that account for historical exposure and the relationship between clinical malaria and infection parasite count. To investigate the strength of this association, one approach would be including the parasite countas a time-dependent covariate in the model of time-to-clinical malaria episode. However, this approach may be inaccurate because it does not account for the fact that parasite count in this case is an endogenous covariate whose existence and future path can be directly related to the occurrence of the episodes [6]. Standard time-to-event models require that time-dependent covariates be external to the event process [7] but clinical malaria disease frequently leads to treatment which may instantly affect the level of parasite count.

A second approach is to fit a joint model of time-to-clinical malaria and longitudinal parasite count profile. Previous applications of the joint modelling framework are many. These include, for example, analysis of CD4 count jointly with time-to-development of AIDS [8–12] and modelling quality of life performance scores jointly with time-to-death or disease progression among patients with cancer [11,13,14]. In order to fit joint models to data from malaria studies, there are certain aspects of these types of data that require consideration. In particular, following treatment it is possible for the parasite count to equal zero, so the joint model should allow for that.

We investigated whether modelling time-to-new clinical malaria disease jointly with parasite count trajectory data may improve precision in log hazard ratio estimates when compared to the conventional Cox PH model with time-dependent parasite count. We also assessed the strength of the association between the hazard of clinical malaria and a time-varying parasite count.

## Methodology

### Data source

The study was motivated by data from the Mfera prospective cohort study conducted in Chikwawa district, southern Malawi, described previously by Buchwald and others [15]. Malaria disease is endemic in Malawi [16] and transmission of the *Plasmodium falciparum* parasite is high in Chikwawa [17,18]. The cohort enrolled 120 participants who presented with uncomplicated malaria at the Mfera health centre in the Chikwawa district between June 2014 and March 2015. Initial diagnosis was made by rapid diagnostic test (RDT) and confirmed by microscopy using thick blood smears. Exclusion criteria from the Mfera cohort included: acute illness requiring hospitalization, signs or symptoms of severe malaria or moderate to severe anemia, and chronic medication with any drug that has antimalarial activity e.g. HIV treatment. Participants underwent passive and active surveillance on a monthly basis and whenever sick for up to two years to assess re-infection, host response and parasite count.

### Primary outcome

In the current analyses, the outcome of interest was time-to-first new clinical malaria disease which was defined by participants’ self-reported fever and apositive RDT result.

### Notation and specification of the models

The conventional Cox PH model with time-dependent parasite count is defined as in [9] with the hazard function *λ*_*i*_*(t)* for participant at given time expressed as.

(1) $$\lambda_{i}\left(t\right)=\lambda_{0}(t)\exp\left[\beta_{s}X_{si}^{'}\left(t\right)\right],$$

where and *λ*_*0*_*(t)* is the unspecified baseline hazard function and the covariate vector *X*_*si*_*(t)* for participant includes participant’s age and frequency of insecticide treated bed nets use in the previous month of the visit. Covariates were included in multivariable models if they were significant at alpha level of 0.1 in univariate Cox PH models.

For the joint models, we utilised the Bayesian joint modelling approach for longitudinal and survival data proposed by Chen et al [12] which fits a model with Markov Chain Monte Carlo (MCMC) methods as presented by Ibrahim et al [19]. The joint model is composed of longitudinal and survival sub-models. The longitudinal sub-model takes the form of a mixed-effects model as follows; supposing data is available from *N* participants with *n*_*i*_ observations recorded for participant *i, (i=1,……,N)*. The response *y*_*ij*_ *(j=1,…….,n*_*i*_*),* fixed-effect covariate vector *X*_*ij*_*=(X*_*1ij*_ *,…….,X*_*pij*_*)’*, and random-effect covariate vector *Z*_*ij*_*=(Z*_*1ij*_ *,…….,Z*_*qij*_*)’* are recorded at times *t*_*ij*_. The longitudinal sub-model is

(2) $$y_{ij}=\beta X_{ij}^{'}+b_{i}Z_{ij}^{'}+\varepsilon_{ij},$$

where *β* is the *p×1* fixed-effect parameter vector, *b*_*i*_ is the *q×1* vector of random effects for participant which is assumed to be multivariate normal with mean zero, i.e., *b*_*i*_*~N*_*q*_ *(0,∑*_*b*_*)*, and *∑*_*b*_ is the variance-covariance matrix of the subject specific effects. The error vector $\varepsilon_{i}={\left(\varepsilon_{il},..,\varepsilon_{n\;j}^{\;\;i}\right)}^{'}$ is assumed to be distributed $\varepsilon_{i}\sim N_{n}^{i}\left(0,\delta^{2}l_{ni}\right)$ where *δ*^*2*^ is variance and *I*_*ni*_ is the *n*_*i*_*× n*_*i*_ identity matrix.

The survival sub-model takes a Cox PH model form [9] where the hazard function for *λi (t)* participant *i* at time *t* as given in equation (1) is modelled as

(3) $$\lambda_{i}\left(t\right)=\lambda_{0}\left(t\right)\exp\left[\theta h\left(\beta ,b_{i},t\right)+\beta_{s}X_{si}^{'}(t)\right],$$

where *h(β,b*_*i*_*,t)* is a function of the fixed and random effects in the longitudinal sub-model, and *θ* is an association parameter linking the two sub-models: survival and longitudinal models. The *β*_*s*_ is a parameter vector for covariates unique to the survival sub-model. The survival covariate vector *X*_*si*_*(t)=(x*_*si1*_*,..,x*_*sir*_*)*’ may include baseline covariates for participant *i* with *β*_*s*_ representing *r×1* parameter vector. The functional form of *h(β,b*_*i*_*,t)* determines the type of the association structure between longitudinal parasite count and the time-to new clinical malaria episode. Taking the first derivative of *h(β,b*_*i*_*,t)* is interpreted in terms of an association of the rate of change in parasite count at time and the hazard of new clinical malaria episode at the same time, while the integral of *h(β,b*_*i*_*,t)* would relate the hazard and the cumulative parasite count trajectory defined as area under parasite count profile from baseline up to the time of the new episode.

Once the two sub-models are specified, the likelihood for the joint model can be constructed as follows. Let *T*_*i*_ and *C*_*i*_ represent potential failure and censoring times respectively for participant *i*. Let *S*_*i*_*=min {T*_*i*_*,C*_*i*_*}* be the minimum of the observed failure and censoring times for participant *i*, and let *τ*_*i*_ be the failure indicator taking value 1 if *T*_*i*_ *< C*_*i*_ and 0 otherwise. Then the value of the longitudinal trajectory for participant *i* at time *t* can be defined as *φ*_*i*_ *(β,b*_*i*_*,t)* and at visit *j* as *φ*_*ij*_ *(β,b*_*i*_*)*. Using the full longitudinal trajectory, then the likelihood of the joint distribution of the observed data and random effects for participant can be decomposed as

(4) $$L_{i}\propto f_{i}\left(Survival|longitudinal\right)\times f_{i}\left(longitidinal\right)\;=f\left(S_{i}|\theta ,\tau_{i},\beta_{s},\varphi_{i}\left(\beta ,b_{i},t\right),X_{si}\right)\times f\left(y_{i}|x_{i},z_{i},\beta ,b_{i}\right)f\left(b_{i}\right)$$

and the joint likelihood for all participants can be written as $L={\prod_{i=1}}^{N}L_{i=1}$.

Denoting parasite count as PC, the likelihood of the joint distribution of observed data, random effects and PC can be expressed as

(5) $$L=f(S|\theta ,\tau ,\beta_{s},\varphi \left(\beta ,b,t,PC)\right)\times f\left(b,PC|\beta \right)$$

and integrating out the random effects of the conditional likelihood yields the marginal likelihood. Under the Bayesian framework, the random effects are sampled in MCMC algorithm as extra parameters. The survival and longitudinal sub-models are linked by sharing common random effects structure. The MCMC computations of the model parameters proceed assuming that given the random effects, the longitudinal parasite countand time-to-clinical malaria process are independent as are the longitudinal responses of each participant.

### Data analysis

Baseline data was summarised using frequencies and percentages if categorical and medians with ranges were presented if continuous and skewed. Failure functions for time to new clinical malaria disease were summarised using Kaplan Meier plots, and compared across age and bed net usage using logrank test. Four models of time-to-new clinical malaria disease were fitted. These included the reference model (model 1), a conventional Cox PH model fitted with time-dependent parasite count measured at each visit, and three competing joint models. The three joint models take a common formulation only differing in the assumed association structure linking the hazard of clinical malaria with parasite count. The hazard of clinical malaria disease at any time was linked with: 1) the current underlying value of parasite count at the same time point (model 2), 2) the rate of change in parasite count trajectory at time (model 3), and 3) the cumulative trajectory or area under profile of parasite count from baseline up to time (model 4). Each joint model involved fitting a quasi-Poisson mixed-effects sub-model of parasite count longitudinal trajectory with natural spline functions of time and including the resulting estimates as covariates in the Cox PH sub-model. Other covariates in the conventional Cox PH and survival sub-models included age of the participant and frequency of insecticide treated bed net use in previous month. Parameters were estimated using MCMC through the random walk Metropolis–Hastings (M-H) algorithm. Diffused normal priors were assumed for the covariates including the association parameter. Diagnostic assessments were conducted to assess the convergence of the MCMC samples using trace and kernel density estimator plots, for the final optimal model. Analyses were done in Stata SE version 15.1 (Stata Corp., College Station, TX) [20] using programme *bayesmh* and R version 3.4.3 using packages *JMbayes, survival* and *glmmPQL* [21].

## Results

There were 120 participants in the cohort, of which 69 (57.5%) were females. The overall median age was7.5 years [inter-quartile range (IQR): 4.7–18.1], 6.3 years (IQR: 3.2–13.1) for males and 9.2 years (IQR: 5.3–18.5) females (Table 1). The median number of malarial parasites per μL was 11,060 (IQR: 840–54,000) overall, 24,840 (IQR: 1,600–68,600) in males, and 5,640 (IQR: 520–540, 000) for females. During enrolment, 48 (44.9%) out of 107 particiants reported to have been using bed nets every night in previous month.

### Follow up and time-to-first new clinical malaria disease

Analyses of time-to-new clinical malaria disease included 115 participants who had a least one follow-up visit post enrolment and together contributed a total 894 observations. The median follow-up time to new clinical malaria disease episode was 3.5 months (IQR: 1.1–7.9). Out of the 115 participants, 100 (87.5%) experienced the episode while 15 (12.5%) were administratively right censored (Table 2). Among 100 participants who experienced the episode, 58 (58%) were females, 48 (48%) were aged 5–15 years, 37 (37%) reported using bed net nightly in prior month to enrolment and their median parasite count was 13640 (IQR: 840 – 52040).

### Parameter estimation

Regression coefficient estimates are log hazard ratios, log (HRs). Overall, the joint models gave larger log (HR) estimates with consistently smaller standard errors and narrower credible intervals when compared to the conventional Cox PH model with time-dependent parasite count (Table 3). Comparing the Deviance Information Criteria (DIC) from the three joint models showed that joint model 4 with cumulative parasite count had the lowest value (Table 4), suggesting the best fit [22]. For the rest of the manuscript, the joint model is referencing to the joint model 4 with cumulative parasite count which is being compared to the conventional Cox PH model (model 1). Considering adults above 15 years as reference group, the log(HR) of clinical malaria disease for children under 5 years,was 0.74 [95% Credible Interval (CI): 0.17, 1.26] in the joint model compared to 0.62(95% CI: 0.04, 1.18) from the conventional Cox PH model. The log (HR) for participants aged 5–15 years was 0.72 (95% CI: 0.22, 1.22) in the joint model compared to 0.63(95% CI: 0.11, 1.17) in the conventional Cox PH model. Considering participants who used a bed net nightly in the previous month as reference, the log (HR) of clinical malaria disease for those who did not use a bed net every night was 0.58 (95% CI: 0.13, 1.07) from the joint model compared to 0.52 (95% CI: 0.07, 1.08) in the conventional Cox PH model. From the joint model, the area under the longitudinal trajectory of parasite count was strongly associated with the risk of clinical malaria, with a unit increase corresponding to a −0.0012 (95% CI: −0.0021, −0.0004) decrease in the log hazard ratio. Neither current underlying value of parasite count nor rate of change in parasite count trajectory at the same time point were significantly associated with the occurrence of a new clinical malaria episode.

### MCMC convergence of estimated log (HR) parameters from final optimal joint model

The MCMC sampler trace plots from the final joint model4 seemed to mix well for parameters of children under 5 years, 5–15 years and infrequent bed net use and never moved beyond 1.5 achieving convergence (Figure 2A–2C). The sampler for cumulative parasite count parameter, showed noisy pattern before converging at about 1000 iterations (Figure 2D). From the Kernel density estimator plots, all the four parameters were roughly normal suggesting that the M-H algorithm sampled within the target normal distribution (Figure 3A–3D).

### Factors associated with risk of new clinical malaria episode

**Figure 1:**
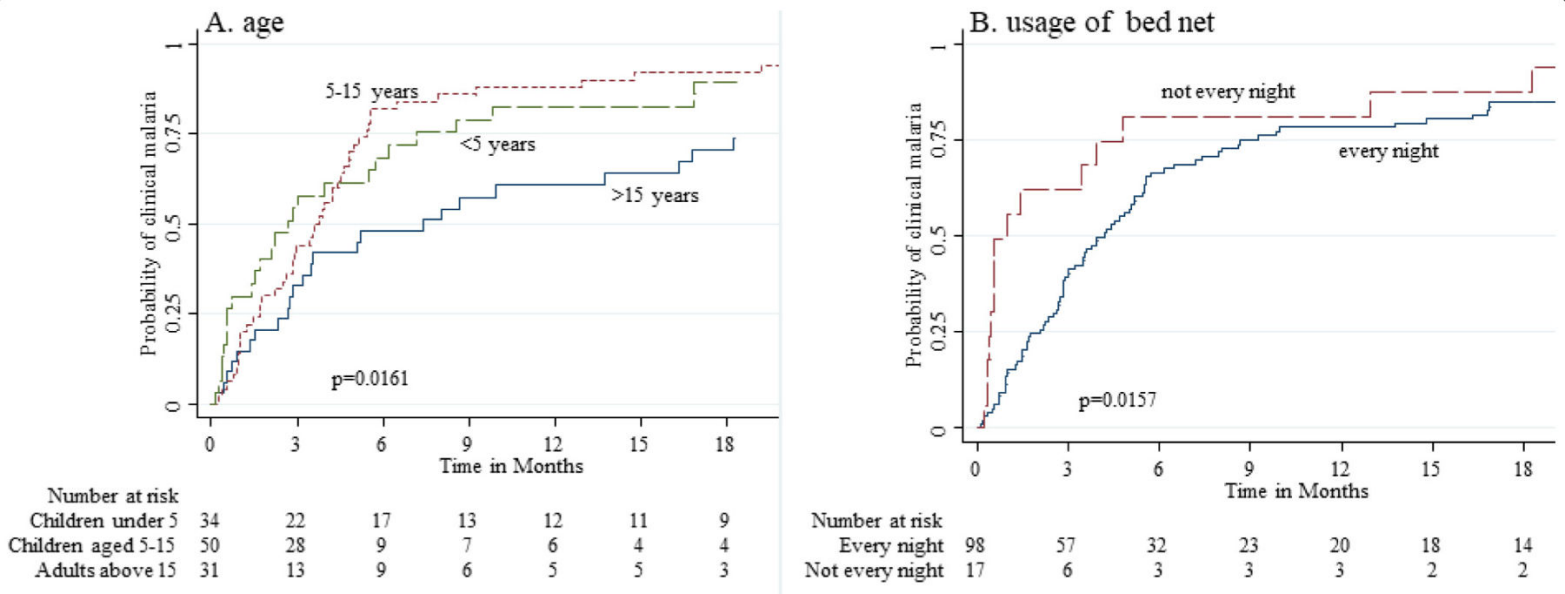
Kaplan-Meier estimator for time-to-new clinical malaria episode by **A**) baseline age, and **B**) use of bed net in previous month. The risk of experiencing clinical malaria episode was significantly higher in children under 5 years and of 5–15 years, and among participants who did not use bed nets every night.

**Figure 2:**
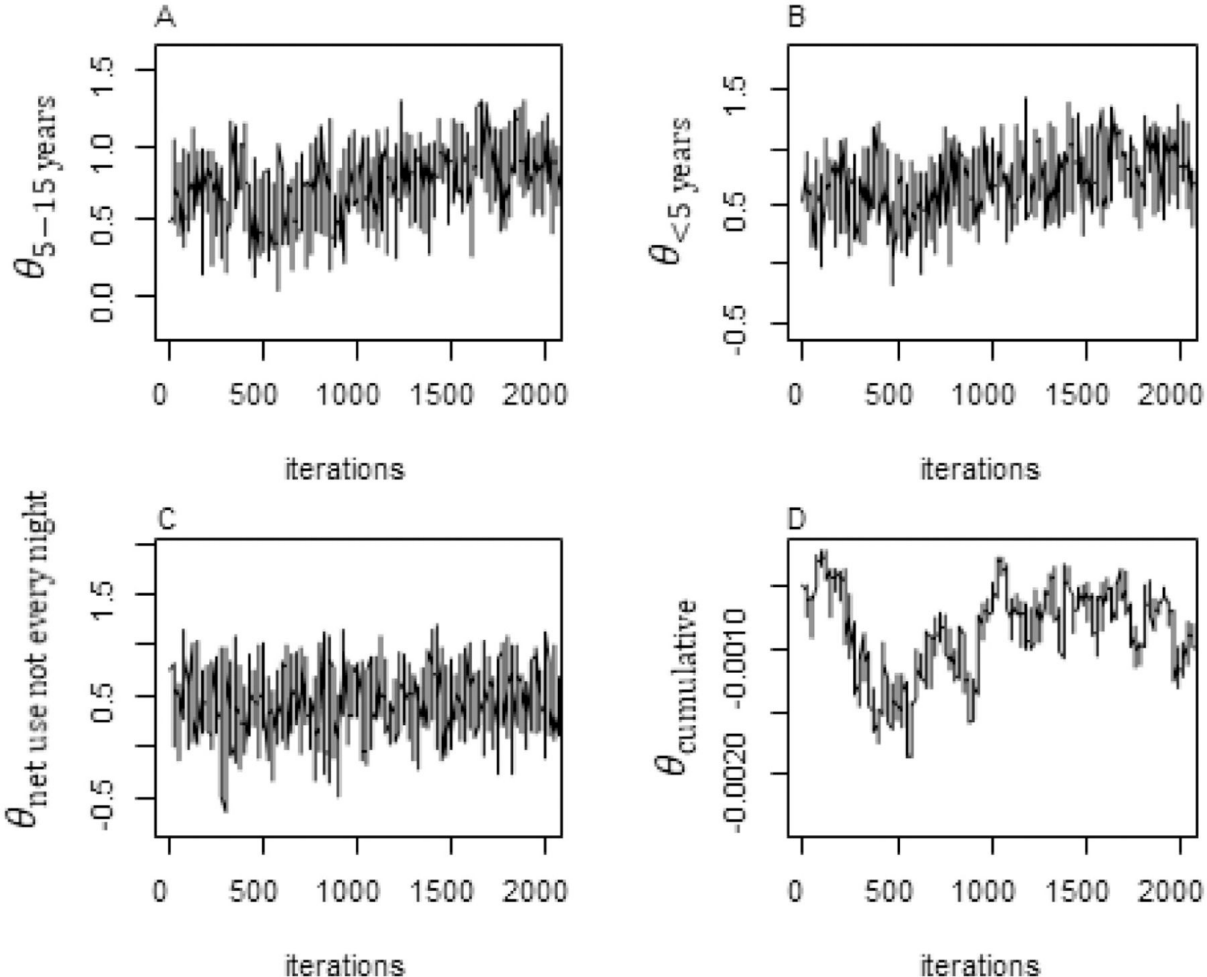
Trace plots for parameters from final joint model. Trace plots show values that the parameter took during the runtime of the MCMC sampling until it reached convergence. Trace plots for parameters *θ*_*5–15 years*_*, θ*_*<5 year*s_ and *θ*_*net use not every night*_ show that the M-H samplers explored the distribution by traversing to areas where its density is very low with very small fluctuations, suggesting that the chains mixed well to the target distributions. In D, the sampling for the parameter of cumulative parasite count *θ*_*cumulative*_ shows noisy pattern before converging at about 1000 iterations.

**Figure 3:**
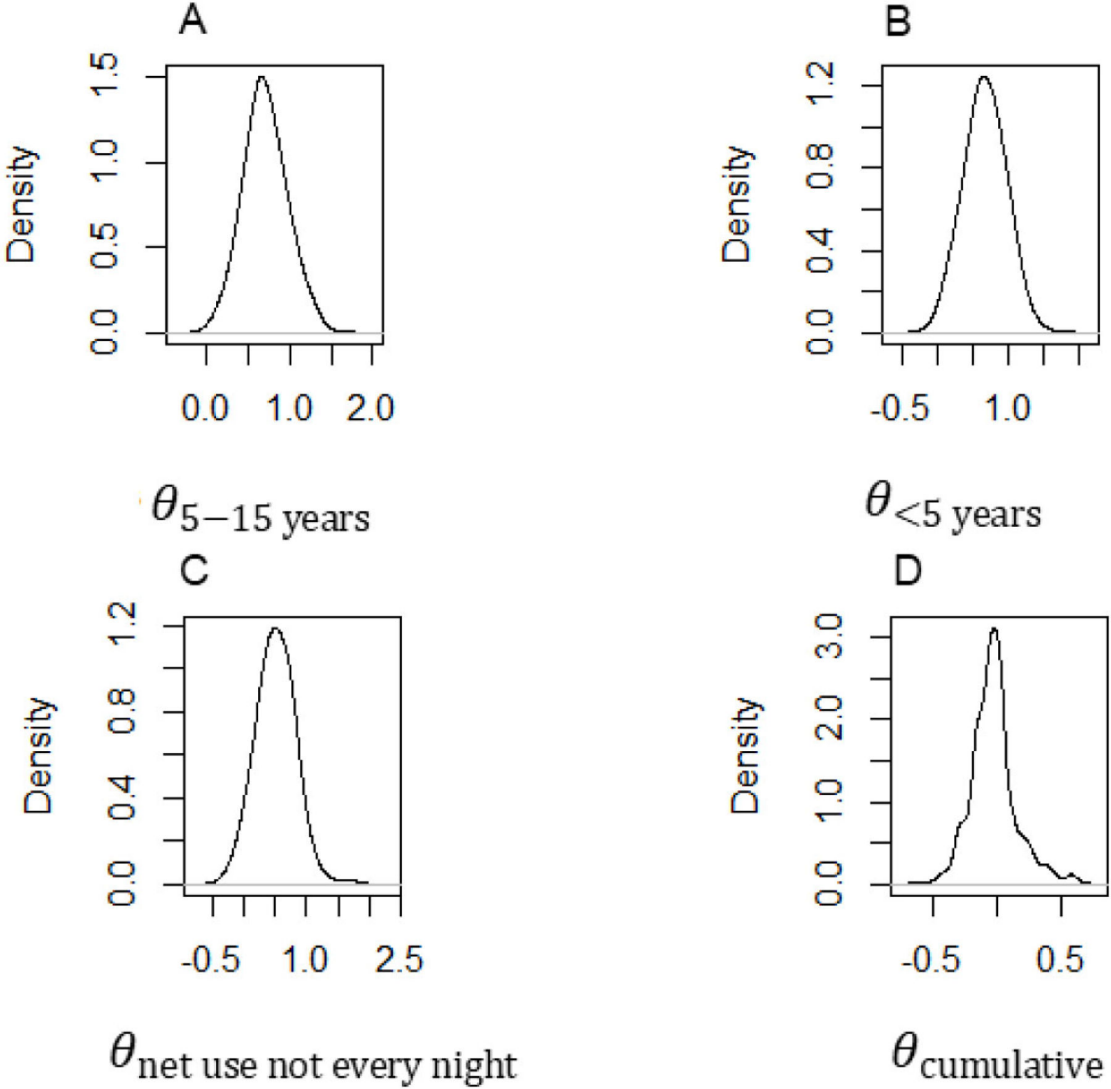
Kernel density estimator plots for the parameters of the final joint model. The MCMC sampling process of the parameters portrays the target poster distribution. The plots suggest that algorithm sampled successfully within the assumed normal distribution for all parameters *θ*_*5–15 years*_, *θ*_*<5 year*s_, *θ*_*net use not every night*_ and *θ*_*cumulative*_.

In unadjusted analyses using Kaplan Meier failure estimator, the risk of experiencing new clinical malaria episode was higher in children under 5 years and the 5–15 years compared to adults above 15 years (log-rank p-value=0.016) (Figure 1A). The risk of getting a new clinical malaria episode was also higher in participants who did not use bed net every night compared to those who used a bed net nightly in previous month (log-rank p-value=0.016) (Figure 1B). Based on the joint model, conditional on cumulative parasite count profile, the hazard of getting new clinical malaria episode was higher in children under 5 years by 2.1-fold (95% CI: 1.2, 3.4) and those aged 5–15 years by 2.0-fold (95% CI: 1.2, 3.5) when compared to adults over 15 years old and controlling for bed net usage. Among participants of the same age, the conditional hazard was also higher for participants who did not use a bed net every night by 1.8 -fold (95% CI: 1.1,3.3) compared to those who used the bed net nightly in previous month.

## Discussion

This study has demonstrated that jointly modelling longitudinal parasite count and time-to clinical malaria episodes improves precision in risk factor estimates associated with clinical malaria disease. The joint model yielded larger parameter estimates with consistently smaller standard errors and narrower credible intervals when compared to a conventional Cox PH model with time-dependent parasite count. These results are consistent with findings from other areas including HIV [8,12] and cancer [13,14,23 ] where joint modelling out-performs separate analyses in terms of optimal use of the available information giving both more precise and less biased estimates. The improved precision provided by the joint model may improve study efficiency, for example, clinical trials may be designed with relatively lower sample sizes while still yielding high power. In general, the conventional time-dependent Cox PH model like any other standard time-to-event model assume that time-dependent covariates are external to the event process [7]. However, parasite count in this case is an endogenous covariate whose existence and future path can be directly related to the occurrence of the malaria episodes. By postulating a model for the joint distribution of the covariate parasite count and the time-to-malaria processes, the joint model explicitly accounts for possible inter-dependence between the two processes through shared random effects [24]. Moreover, the time-dependent covariate model assumes that the value of the parasite count does not change until a new measurement is taken which may not be correct. When we modelled parasite count using a quasi-Poisson mixed effects model, we were creating a model for the outcome at any time-point there by indirectly addressing the measurement error. The joint model also offered flexibility to investigate the appropriate link structure between longitudinal parasite count and time-to-new clinical malaria episode. Among the fitted joint models, only the model with cumulative parasite count was strongly associated with the hazard of new clinical malaria episode suggesting that the risk can better be explained by conditioning on the cumulative effect of the longitudinal parasite count trajectory from baseline up to the episode time. The underlying current value of the parasite count or change in parasite count at any time was not associated with the risk of experiencing new clinical malaria episode at the same.

Factors associated with a high risk of experiencing a new clinical malaria episode were young age and infrequent use of bed nets as have been reported in other studies [25–28]. Children aged up to 15 years had higher risk for clinical malaria disease when compared to adults over 15 years. The higher risk of experiencing a clinical malaria episode among children is possibly be due to the naturally under-developed humoral immune responses to different stage-specific antigens of *P. Falciparum* otherwise acquired with age [29]. In this study in an endemic area, high cumulative parasite count was associated with lower risk of getting a new clinical malaria episode suggesting that increased exposure to malaria parasites with time may result into protective effect to future clinical malaria episodes.

This study may be limited by focusing on time-to-first malaria episode only, thus estimates obtained here may not be applicable to analyses examining all clinical malaria episodes over a follow up period. This paper has established the optimal way of incorporating parasite count in estimating time-to-first new clinical malaria which may further be extended to study recurrent episodes over entire follow-up, but this may require different distributional assumptions. There were missing values for bed net use which may have affected the findings. Future studies should include multiple imputation of missing covariate data and also investigate the role of measurement error due to detection limit of parasite count in these joint models. Further work is required to consider joint modelling of parasite count with recurrent episodes and to predict risk of future clinical malaria episodes.

## Conclusions

In conclusion, jointly modelling longitudinal parasite count and time-to-new clinical malaria improved precision in log hazard ratio estimates for clinical malaria when compared with the conventional Cox PH model with time-dependent parasite count. The improved precision of joint modelling may improve study efficiency and allow for design of clinical trials with relatively lower sample sizes with increased power.

## Figures and Tables

**Table 1.** Baseline demographic, clinical conditions and vital signs for Mfera malaria cohort in Malawi.

Variable	Total (n=120)
Gender, female, n (%)	69 (57.4)

Age, n (%)	

< 5 years	34 (28.3)
5–15 years	51 (42.5)
>15 years	35 (29.2)
Weight (kg), median (IQR)	21.5 (15.0 – 46.0)
Height (cm), median (IQR)	119.5 (103.0 – 151.8)
Temperature (°C), median (IQR)	36.7 (36.2 – 38.6)
Respiratory rate (breaths/minute), median (IQR)	28 (22 – 36)
Heart rate (beats/minute), median (IQR)	112 (92 – 139)
Haemoglobin (g/dl), median (IQR)	11.5 (10.2 – 12.4)
Parasite count (number of parasites/µL), median (IQR)	11060 (840 – 54000)
Cough, n (%)	15 (12.5)
Musculoskeletal pain, n (%)	40 (33.3)
Headache, n (%)	36 (30.0)
Vomiting, n (%)	32 (26.7)
Abdominal pain, n (%)	15 (12.5)

Bed net use previous month*, n(%)	
Every night	48 (44.9)
Most nights (> half)	13 (12.1)
Some nights (< half)	8 (7.5)
No nights	38 (35.5)

**Season enrolled, n(%)**	
Dry: May - November	91 (75.8)
Rainy: December - April	29 (24.2)

* not adding up to column total due to missing

**Table 2.** Characteristics by clinical malaria statusfor Mfera malaria cohort in Malawi.

Variable	Clinical malaria episode (n=100)	No clinical malaria episode (n=15)
**Sex, n (%)**		

Male	42 (42.0)	8 (53.3)
Female	58 (58.0)	7 (47.7)
Age, n (%)		
<5 years	27 (27.0)	4 (26.7)
5–15 years	48 (48.0)	2 (13.3)
>15 years	25 (25.0)	9 (60.0)

**Net use previous month, n(%)**		
Every night	37 (37.0)	8 (53.3)
Not every night	63 (63.0)	7 (46.7)
Haemoglobin (g/dl)	11.4 (10.0 – 12.3)	12.3 (10.9 – 13.9)
Parasite count, number of parasites per µL, median (IQR)	13640 (840 – 52040)	2800 (560 – 60040)

Note: clinical malaria status data available for 115 participants who had a least one follow-up visit.

**Table 3.** Log of hazard ratio estimates for time-to-new clinical malaria episode for Mfera cohort in Malawi.

		Age at baseline*		Bed net use in previous month^+^	Parasite count
Model	**Parameter**	*θ_<5 years_*	*θ_5–15 years_*	*θ_not every night_*	*θ_parasite count_*

Model 1	Estimate	0.624	0.632	0.520	−1.77e-5
	Std error	0.282	0.259	0.292	1.86e-6
	95% CI	0.044, 1.183	0.115, 1.174	0.076, 1.085	−1.41e-5, 2.14e-5

Model 2	Estimate	0.672	0.707	0.582	−0.029
	Std error	0.016	0.014	0.024	0.053
	95% CI	0.094, 1.251	0.213, 1.265	0.110, 1.017	−0.345, 0.411

Model 3	Estimate	0.749	0.775	0.594	−0.103
	Std error	0.023	0.021	0.023	0.071
	95% CI	0.077, 1.485	0.193, 1.253	0.159, 1.156	−6.168, 6.124

Model 4	Estimate	0.735	0.722	0.575	−0.001
	Std error	0.022	0.028	0.030	0.0001
	95% CI	0.170, 1.263	0.219, 1.215	0.125, 1.066	−0.0021, −0.0004

Log hazard ratio estimates are posterior means. All models are multivariable. CI= Credible Interval.

* reference: >15 years

+ reference: bed net use nightly.

**Table 4.** Deviance Information Criteria (DIC) from the competing joint models.

Joint Model	DIC
Model 2: Joint model with current underlying value of parasite count at any time	74763360
Model 3: Joint model with rate of change in parasite count trajectory at any time	74763436
Model 4: Joint model with cumulative parasite count from baseline up to any time	74763318
